# Expression profiling and cross-species RNA interference (RNAi) of desiccation-induced transcripts in the anhydrobiotic nematode *Aphelenchus avenae*

**DOI:** 10.1186/1471-2199-11-6

**Published:** 2010-01-19

**Authors:** Wesley Reardon, Sohini Chakrabortee, Tiago Campos Pereira, Trevor Tyson, Matthew C Banton, Katharine M Dolan, Bridget A Culleton, Michael J Wise, Ann M Burnell, Alan Tunnacliffe

**Affiliations:** 1 Department of Biology, National University of Ireland, Maynooth, Co. Kildare, Ireland; 2 Institute of Biotechnology, Department of Chemical Engineering and Biotechnology, University of Cambridge, Tennis Court Road, Cambridge CB2 1QT, UK; 3 Department of Biology, FFCLRP, University of Sao Paulo, 14040-901, Brazil; 4 Applied Biosystems, Lingley House, 120 Birchwood Boulevard, Warrington, Cheshire, WA3 7QH, UK; 5 School of Biomedical and Chemical Sciences, University of Western Australia, Crawley WA 6009, Australia

## Abstract

**Background:**

Some organisms can survive extreme desiccation by entering a state of suspended animation known as anhydrobiosis. The free-living mycophagous nematode *Aphelenchus avenae *can be induced to enter anhydrobiosis by pre-exposure to moderate reductions in relative humidity (RH) prior to extreme desiccation. This preconditioning phase is thought to allow modification of the transcriptome by activation of genes required for desiccation tolerance.

**Results:**

To identify such genes, a panel of expressed sequence tags (ESTs) enriched for sequences upregulated in *A. avenae *during preconditioning was created. A subset of 30 genes with significant matches in databases, together with a number of apparently novel sequences, were chosen for further study. Several of the recognisable genes are associated with water stress, encoding, for example, two new hydrophilic proteins related to the late embryogenesis abundant (LEA) protein family. Expression studies confirmed EST panel members to be upregulated by evaporative water loss, and the majority of genes was also induced by osmotic stress and cold, but rather fewer by heat. We attempted to use RNA interference (RNAi) to demonstrate the importance of this gene set for anhydrobiosis, but found *A. avenae *to be recalcitrant with the techniques used. Instead, therefore, we developed a cross-species RNAi procedure using *A. avenae *sequences in another anhydrobiotic nematode, *Panagrolaimus superbus*, which is amenable to gene silencing. Of 20 *A. avenae *ESTs screened, a significant reduction in survival of desiccation in treated *P. superbus *populations was observed with two sequences, one of which was novel, while the other encoded a glutathione peroxidase. To confirm a role for glutathione peroxidases in anhydrobiosis, RNAi with cognate sequences from *P. superbus *was performed and was also shown to reduce desiccation tolerance in this species.

**Conclusions:**

This study has identified and characterised the expression profiles of members of the anhydrobiotic gene set in *A. avenae*. It also demonstrates the potential of RNAi for the analysis of anhydrobiosis and provides the first genetic data to underline the importance of effective antioxidant systems in metazoan desiccation tolerance.

## Background

Water is essential for life, but some organisms have the ability to survive extreme desiccation by entering a state of suspended animation in a process known as anhydrobiosis ("life without water") [1]. In the dry state, metabolism and life processes come to a halt. Anhydrobiotic organisms are capable of surviving in this state for an indefinite period of time after which, when environmental conditions become favourable, the organism can resume normal metabolic activity [2,3]. These organisms are widespread throughout nature and include yeasts, bdelloid rotifers, tardigrades, plants and nematodes. For example, the resurrection plant *Craterostigma plantagineum *can recover from loss of most of its cellular fluid within 24 h of contact with water [4,5]. Many nematode species are also able to enter anhydrobiosis, for example, *Aphelenchus avenae *and *Panagrolaimus superbus *[6,7]. Some steinernemitid species also exhibit partial desiccation tolerance [8,9].

Anhydrobiosis is often associated with the accumulation of disaccharides and other oligosaccharides, chiefly trehalose in animals and yeasts, and sucrose in plants [1,4,10]. These sugars have been suggested to play a role in protecting membranes and proteins by replacing structural water or by forming a stabilising intracellular glass that would inhibit membrane fusion and protein denaturation; they might also be involved in maintaining membrane fluidity [11,12]. However, it has been shown in *A. avenae*, one of the best-characterised anhydrobiotic nematodes, that while it accumulates trehalose prior to anhydrobiosis, probably at least partly due to upregulation of trehalose synthase genes [13], this alone is not sufficient [14]. In other desiccation-tolerant invertebrates, such as bdelloid rotifers [15-17] and some tardigrades [18], disaccharide accumulation is not detectable. In the yeast *Saccharomyces cerevisiae*, trehalose biosynthesis can be abolished by mutation while retaining 50-100% capacity to survive desiccation, depending on the strain [19]. This suggests that while trehalose might play a role in anhydrobiosis, at least in some species, there must be other important biological processes involved [20]. These processes might include changes in primary metabolism; alterations to cell membranes; the accumulation of compatible solutes and hydrophilic proteins; and activation of antioxidant and molecular chaperone systems.

One group of proteins receiving increasing attention is the LEA (late embryogenesis abundant) proteins. These were first associated with desiccation tolerance in plant seeds and resurrection plants (reviewed in [21]), but recently homologues of Group 3 LEA proteins have been linked to anhydrobiosis in certain nematodes [22-25] and other invertebrates [17,26-29]. Group 3 LEA proteins are characterized by extreme hydrophilicity and 11-amino acid periodicity; they have relatively little secondary structure in the hydrated state, but can become more structured on drying [21,30]. A nematode Group 3 LEA protein from *A. avenae*, AavLEA1, has been shown to function as a protein anti-aggregant both *in vitro *and *in vivo *[31,32]. It has therefore been postulated that many LEA proteins possess a molecular shield activity whereby they reduce inappropriate interactions between other proteins by an electrosteric mechanism [31,33]. LEA proteins also exhibit other activities consistent with a role in protection of cells against abiotic stress, including membrane protection, ion binding, antioxidant functions, hydration buffering and nucleic acid binding (references in [21] and [34]).

In this study we have constructed an EST (expressed sequence tag) library of genes upregulated on desiccation in the free-living mycophagous nematode *A. avenae *and have investigated their expression profiles under various stress conditions. We have identified a new LEA protein gene, as well as other stress response genes, and have used a cross-species gene silencing approach to assess the importance of a subset of the EST panel for anhydrobiosis. Our findings indicate that glutathione peroxidases represent a key class of enzymes required for desiccation tolerance in the nematodes tested and emphasise the need for redox balancing in anhydrobiosis.

## Results

### An EST dataset from *A. avenae *enriched for dehydration-induced sequences

A 5' oligo capping method was used to construct an EST library of genes enriched in those upregulated on drying in *A. avenae*: an initial screen of 984 clones by reverse Northern dot blots, using cDNA from both dried and control nematodes in parallel experiments, yielded 88 ESTs which appeared to be induced by desiccation. From these, a subset of 30 ESTs showing significant BLAST matches to sequences from other organisms, and 12 ESTs representing apparently novel sequences, were selected for further study (Table 1).

**Table 1:** ESTs, with similarity to known sequences, identified by enrichment for dehydration-induced gene expression in *A. avenae*.

Relative Expression							
**Accession no.**	**Closest BLAST hit (organism)**^a^	**Homologue accession no. **	**BLAST score**	**Desiccation**	**Osmotic**	**Heat**	**Cold**

EF026241	LEA protein (*Caenorhabditis elegans*)	NM_001028871	0.001	42.6	24.1	3.1	7.9
EF026240	*LEA-like hypothetical protein (*C. elegans*)	AF016513	0.002	35.9	21.8	2.8	47.2
GR463894	C-type lectin (*clec-49*) (*C. elegans*)	NM_075428	2E-12	1.7	0.7	1.3	1.1
EF026242	*C-type lectin (*clec-202*) (*C. elegans*)	NM_070694	2E-09	1.3	2.4	1.5	1.9
GR463895	polygalacturonase (*Aspergillus clavatus*)	XM_001272238	5E-51	2.7	47.9	0.9	7.2
GR463896	polygalacturonase (*Penicillium griseorseum*)	AF195791	1E-129	3.0	2.1	0.7	3.2
GR463897	AHA-1, activator of Hsp90 (*Bombyx mori*) *myo*-inositol-1-phosphate synthase (*C. elegans*)	XM_001901615	2E-17	27.2	19.3	1.7	4.3
GR463898		NM_064098	8E-68	1.3	1.3	0.9	1.6
GR463899	GABA/glycine receptor (*ggr-3*) (*C. elegans*)	NM_062550	5E-42	1.2	15.3	1.6	0.8
GR463900	hypothetical protein Y53G8B.4 (*C. elegans*) C2H2-type zinc finger protein (*Brugia malayi*)	NM_001047419	3E-16	4.4	12.5	4.5	6.2
GR463901		XM_001900365	1E-104	2.6	5.2	2.7	3.5
GR463902	*MSFI-like/14-3-3 protein (*C. elegans*)	NM_060551	1E-65	2.6	2.5	1.0	2.0
DR121003	vesicle transport (*unc-16*) (*C. elegans*)	NM_001027810	2E-09	2.4	1.7	1.2	1.6
GR463903	DJ-1 like (*Tribolium castaneum*)	XM_968208	4E-37	10.2	6.3	2.0	3.6
GR463904	*saposin (*spp-3*) (*C. elegans*)	NM_076834	4E-05	1.8	2.0	1.8	3.0
GR463905	Sm-like protein (*lsm-5*) (*C. elegans*)	NM_074469	1E-42	4.3	2.9	1.0	2.9
GR463906	dual specificity phosphatase F28C6.8 (*C. elegans*)	NM_063425	3E-28	2.8	2.3	1.3	2.7
GR463907	Appr-1-p processing enzyme (*B. malayi*)	XM_001901533	5E-21	1.3	3.1	1.7	1.9
GR463908	hypothetical protein F42A10.7 (*C. elegans*)	NM_065940	1E-41	1.8	1.7	1.3	2.6
GR463909	aspartate protease inhibitor (*Onchocercavolvulus*)	X13313	4E-35	2.5	4.9	2.7	8.2
GR463910	GATA-type zinc finger protein (*B. malayi*)	XM_001892676	6E-14	2.6	1.9	1.4	2.0
GR463911	tropomyosin (*Heterodera glycines*)	AF546757	1E-99	1.6	1.7	1.9	2.5
GR463912	transthyretin-related (*Radopholus similis*)	AM691117	2E-36	4.3	2.9	0.7	5.6
GR463913	collagen family (*col-14*) (*C. elegans*)	NM_001047459	1E-39	1.4	1.4	1.3	2.6
GR463914	glutathione peroxidase (*Globodera rostochiensis*)	AJ493678	3E-56	31.9	14.3	1.6	7.6
GR463915	high mobility group protein (*hmg1.1*) (*C. elegans*)	XM_001893327	3E-35	4.0	10.2	4.4	13.4
DR121009	zinc/RING finger protein (*mnat-1*) (*C. elegans*)	NM_061879	3E-14	1.4	2.3	1.1	1.5
GR463916	Predicted ion channel (*ced-11*) (*C. elegans*)	AJ272503	2E-06	1.1	17.6	2.1	0.7
GR463917	rhoGAP domain (*B. malayi*)	XM_001894857	6E-36	2.2	1.5	1.4	0.8
GR463918	hypothetical protein ZK228.3 (*C. elegans*)	Z82086.1	4E-04	1.2	2.0	1.4	2.0

Where accession numbers are shown in bold, respective sequences were used in cross-species RNAi experiments with *P. superbus*. ^a^Closest homologue in the GenBank database using the TBLASTX tool http://www.ncbi.nlm.nih.gov/blast; where indicated by an asterix, BLASTX against the *C. elegans *genome was used instead http://www.wormbase.org/db/searches/blast_blat. Relative expression following exposure of nematodes to four environmental stresses is shown at right of Table. Values in regular type represent a statistically significant upregulation in gene expression with respect to *A. avenae *5.8S rRNA and *ama-1 *genes; values in grey font are not significant.

**Figure 1:**
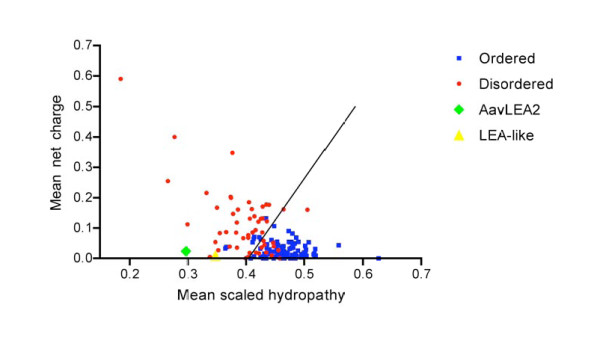
**Uversky plots (mean scaled hydropathy, <*H*>, against mean net charge, <*R*>, at neutral pH) as a predictor of disordered protein structure**. Predicted protein sequences corresponding to AavLEA2 (EF026241) and LEA-like protein (EF026240) were analysed at http://www.pondr.com. The positions of test sequences are shown by a green diamond and a yellow triangle, respectively, in comparison to a set of disordered proteins (red circles) and a set of ordered proteins (blue squares). The boundary line shown between unfolded and folded space is empirically defined by the equation <*H*>_*b *_= (<*R*> + 1.151)/2.785 [109].

The ESTs include a novel homologue of known Group 3 LEA protein genes: this cDNA sequence (accession number EF026241) is full length, as indicated by the presence of a spliced leader sequence (SL1c) at its 5' end and a polyA tail at the 3' end. The cDNA is 405 bp long, excluding the polyA tail, and its longest open reading frame predicts a protein of 85 amino acids with Mr 9990 and pI 6.4. The closest BLASTX match is with *lea-1 *of *Caenorhabditis elegans *and, although the BLAST score is not highly significant (0.001), other features of the predicted protein sequence identify it as a Group 3 LEA protein. For example, the predicted protein sequence has a section of K11 periodicity, with two interlaced runs of three lysines at residues 38, 40, 49, 51, 60 and 62; POPP analysis [35] places it cleanly within the Group 3 LEA protein cluster; it lacks cysteine and tryptophan residues; it is hydrophilic along its entire length according to a hydropathy plot based on the Eisenberg Consensus scale, with a grand average hydropathy (GRAVY) score of -1.83 indicating extreme hydrophilicity (the average score for Group 3 LEA proteins is -0.97; [36]). Predictors of protein disorder suggest the protein is natively unfolded: the VL-XT algorithm at pondr.com indicates 95% disorder, while Foldindex and IUPred both return predictions of 100% disorder; and a Uversky plot of mean net charge against mean scaled hydropathy places the protein firmly among the set of intrinsically disordered proteins (Figure 1). All the above properties are consistent with this example being a new Group 3 LEA protein and therefore we designate it AavLEA2 (as the second such protein from *A. avenae *after AavLEA1; [24]) and its gene *Aav-lea-2*.

A second example from the EST dataset, EF026240, also encodes a hydrophilic protein but it is less clearly identifiable as an LEA protein. The cDNA is again full length, with spliced leader (SL1a) and polyA tail, whose 464 bp insert encodes a protein of 102 amino acids in its longest open reading frame, with Mr 11656 and pI 6.53. The protein has a GRAVY score of -1.38, and a hydropathy plot shows it to be hydrophilic along almost its entire length. Disorder prediction algorithms are consistent with it being unfolded - VL-XT, Foldindex and IUPred predict 78%, 100% and 100% disorder respectively - and the Uversky plot positions the protein within natively unfolded space (Figure 1). POPP analysis shows clustering with Group 2 and Group 3 LEA proteins, but only at the level of band 3, indicating that this association is rather weak. Furthermore, there is no K11, and limited A11, periodicity and the predicted protein sequence includes one cysteine and one tryptophan, which it is unusual to find in Group 3 LEA proteins. A BLASTX run against the *C. elegans *genome returns *lea-1 *as the top hit, although the score is weak (0.002); a BLAST enquiry against the whole sequence collection returns as best hit (0.029) an LEA-like sequence from the Antarctic bacterium *Polaromonas *sp. JS666. Intriguingly, though, using BLAST to compare the EF026240 protein against AavLEA2, described above, gives a score of 5E-14, indicating a match of respectable significance. When aligned, the two predicted protein sequences are given as 34.3% identical with the ALIGN algorithm. In summary, the bioinformatics are consistent with this example being a hydrophilic, intrinsically disordered protein which probably has a distant evolutionary relationship to the Group 3 LEA proteins. For the moment, therefore, we consider it to be an LEA-like hypothetical protein.

While the presence of LEA and LEA-like protein gene sequences among the dehydration-induced EST dataset is in line with a presumed role for these polypeptides in the response to water stress, several other genes associated with stress responses were also found. For example, genes encoding: *myo*-inositol-1-phosphate synthase which controls synthesis of an osmolyte, *myo*-inositol, implicated in desiccation tolerance [37,38]; AHA-1, a stress-regulated activator of the ATPase activity of molecular chaperone Hsp90 [39]; and glutathione peroxidase, which catalyses the reduction of hydroxyperoxides by glutathione and is important in the response to oxidative stress (reviewed in [40]).

The control of the response to desiccation is very poorly understood and therefore it is of interest that a number of genes involved in signal transduction and the regulation of gene activity were recovered from the screen. For example, three zinc finger protein genes were identified (Table 1), encoding a C2H2 zinc finger family member (GR463901), a GATA-type zinc finger (GR463910), and a RING finger domain protein (DR121009). The latter is most likely involved in the regulation of protein activity, rather than gene regulation directly; it gives a best BLAST hit with *mnat-1*, a *C. elegans *gene whose protein is probably involved in the ubiquitination pathway as an E3-type ubiquitin ligase. It is also implicated in stabilising the CDK-activating kinase intrinsic to cell cycle progression through its MAT1 domain and is functionally associated with TFII, the transcription/DNA repair factor (http://www.wormbase.org; [41]). The zinc finger proteins represented by GR463901 and GR463910 would merit further investigation as candidate anhydrobiotic gene set regulators: several transcription factors were found to be upregulated by water loss in *Steinernema feltiae *[8], *S. carpocapsae *[9] and *Plectus murrayi *[25], and a GATA-type transcription factor (ELT-2) has been found to regulate *C. elegans *transcription during the innate immune response and osmotic stress [42]; in plants, many gene regulators have been characterised in the response to drought (e.g. reviewed in [43]). Another gene, represented by GR463906, encodes a dual specificity phosphatase able to hydrolyse phosphate from Tyr and Ser/Thr residues; such phosphatases are key players in signal transduction, for example, in MAPK pathways, and in cell cycle regulation [44]. DR121003 encodes a homologue of *C. elegans *UNC-16 which is primarily involved in vesicle transport, but whose protein is also known to interact with the MAPK, JNK, and also with JNK kinases; it thus functionally intersects with signal transduction pathways. Further investigation of these genes could therefore lead to improved understanding of the dehydration stress response in nematodes.

Twelve of the selected ESTs encode novel proteins that have no matches in the GenBank or WormPep databases (Table 2). Six of these are predicted to be unusually hydrophilic (hydropathy scores <-0.43, the GRAVY score of bovine serum albumin) or largely disordered (>50%) or both, and all are predicted to be charged at physiological pH. (The pHi of resting intestinal cells of *C. elegans *has been determined to be 7.53; [45].) Although they are clearly not LEA proteins, these novel proteins are reminiscent of anhydrin, another highly hydrophilic, disordered protein previously described in *A. avenae*, whose gene expression is induced by drying [24]. The prevalence of such proteins, albeit from a very limited sample, is intriguing and invites further investigation in the context of desiccation tolerance.

**Table 2:** Some predicted physico-chemical parameters of the novel ESTs identified by enrichment for dehydration-induced gene expression in *A. avenae *and their relative expression following exposure of the nematodes to four environmental stresses.

					Relative Expression			
**Accession no.**	**No. amino acids**	**Predicted pI**	**Predicted GRAVY score**	**Percent disorder**^§^	**Desiccation**	**Osmotic**	**Heat**	**Cold**

GR463919	179	9.28	-1.16	75	1.0	1.1	0.6	1.2
GR463920	98	10.51	-0.87	39	1.7	1.8	1.7	1.2
GR463921	88	4.36	0.01	78	1.8	3.4	0.5	1.0
GR463922	168	11.13	-0.55	39	1.5	1.7	1.4	2.1
GR463923	144	6.78	0.20	16	1.2	1.4	1.4	2.2
*GR463924	35	6.69	-0.13	37	4.3	10.9	3.7	8.3
GR463925	143	9.82	-0.08	22	1.5	1.4	1.2	0.5
GR463926	140	9.64	-0.97	51	8.4	4.5	35.7	6.0
GR463927	122	10.91	-0.30	37	1.0	20.6	1.5	0.5
†GR463928	37	6.33	0.28	46	9.0	14.5	0.8	2.2
GR463929	59	4.17	0.99	0	3.5	22.6	4.7	2.7
*EF026246	61	6.35	-2.32	20	1.9	2.2	1.2	2.6

Where accession numbers are shown in bold, respective ESTs were used in cross-species RNAi experiments with *P. superbus*. Expression values in regular type represent a statistically significant upregulation in gene expression with respect to 5.8S rRNA and *ama-1 *genes; values in grey font are not significant. ^§^PONDR VL-XT predictions. *The reading frame selected was from the same strand as the spliced leader sequence. †The reading frame selected was from the same strand as the polyA tail. Where such landmarks were not available the longest predicted open reading frame was used to estimate physico-chemical parameters.

### Evidence for horizontal gene transfer in *A. avenae*

Homologues of two polygalacturonase genes in the fungi *Aspergillus awamori *and *Penicillium griseoroseum *[46,47] were found in the *A. avenae *EST set. These genes seem to be derived from the *A. avenae *genome and are not a result of fungal contamination since preliminary Southern hybridisation experiments show they are not present in *Rhizoctonia solani*, the food source used (data not shown). Polygalacturonases (EC:3.2.1.15) hydrolyse 1,4-alpha-D-galactosiduronic linkages in pectin, a major component of the cell walls of plants [48]. Plant pathogenic bacteria and fungi secrete hydrolytic enzymes such as polygalacturonases and cellulases to degrade plant cell walls. Plant parasitic nematodes also secrete a great diversity of plant cell-wall degrading enzymes including cellulases and galacturonases. Phylogenetic analyses support the interpretation that the cellulases and polygalacturonases present in cyst and root-knot nematodes have been acquired by horizontal gene transfer from bacteria [49-53]. In contrast, the polygalacturonase genes of *A. avenae *appear to be derived by horizontal transfer from fungi (Additional File 1; Table 1). Interestingly a polygalacturonase gene found in the genome of the rice weevil *Sitophilus oryzae *also appears to have been incorporated into the genome of this insect by horizontal gene transfer from a fungal source [54]. While the cellulase and polygalacturonase genes of the root-knot nematode *M. incognita *are most closely related to bacterial sequences, genome-wide analysis indicates that three other plant-cell wall degrading/modifying enzymes in this nematode (pectate lyase, arabinase and expansin) may be of fungal origin [52], and it has also been shown that the cellulases of the pine wood nematode are most similar to cellulases from fungi [55]. *A. avenae *is routinely cultured in the laboratory on fungi such as *Rhizoctonia solani *or *Botrytus cinerea*, but *A. avenae *is also capable of growing and reproducing *in vitro *on tissue cultures prepared from several plant species [56] and it has also been shown that *A. avenae *will feed and multiply on plant roots, and suppress growth [57]. The occurrence of a plant cell wall degrading enzyme in *A. avenae *provides further evidence that in addition to feeding on fungi *A. avenae *might also utilise plant tissues as a food source in nature.

### Multiple variant spliced leaders in *A. avenae *mRNAs

Spliced leaders derive from specific snRNAs and are coupled to pre-mRNAs through a *trans*-splicing mechanism during mRNA maturation [58,59]. Previously, 22-nucleotide spliced leader sequences representing four variants of the SL1 sequence characterised in *C. elegans *have been described at the 5' ends of trehalose-6-phosphate synthase (*tps*) mRNAs in *A. avenae *[13]. All four of these SL1 sequences were found in the ESTs described here, together with eight additional variants (Table 3). Uncovering a total of 12 different sequences from a relatively small sample of mRNA sequences suggests a degree of spliced leader polymorphism in *A. avenae *similar to that reported for *Trichinella spiralis *[60]. Interestingly, four variants (5, 6, 7 and 8) show striking similarity to SL2, the second main form of spliced leader found in *C. elegans*, which is associated with the resolution of polycistronic pre-mRNAs resulting from operon transcription. SL2 is postulated to have first evolved in the rhabditine lineage which includes *C. elegans *[59], but the data of Table 3 suggest that this family of spliced leaders is also present in the Tylenchina, which includes *A. avenae*, and therefore must have arisen no later than the common ancestor of the two sister groups within Nematoda. It will be interesting therefore to investigate whether SL2-like *trans*-splicing is used for processing of operon transcripts in *A. avenae*, like *C. elegans*, instead of SL1, as found in all other nematodes to date outside the Rhabditina [59].

**Table 3:** Twelve different spliced leader sequences in *A. avenae *mRNAs.

SL name	SL sequence	EST accession no.
SL1a	GGTTTATATACCCAAGTTTGAG	EF026240, GR463910, GR463914, GR463901
SL1b	GGTTTTATTACCCAAGTTTGAG	GR463903
SL1c	GGTTTAAATACCCAAATTTGAG	EF026241, GR463900
SL1d	GGTTTAAATACCCTAATTTGAG	GR463894
		
Variant 1	GGTTTAAATACCCTTTATTGAG	GR463919
Variant 2	GGTTTATACACCCAAGTTTGAG	GR463909
Variant 3	GGTTTTATTACCCCAGTTTGAG	GR463912
Variant 4	GGTTTAAATACCCGAATTTGAG	EF026246
Variant 5	GGTTTACACCCAGTATCACAAG	GR463907
Variant 6	GGTTTAATACCCAGTATCACAAG	GR463905, GR463906
Variant 7	GGTTTAAAACCCAGTATCACAAG	GR463915
Variant 8	-GTTTTTACAGAAAACCACAAG	GR463918
		
SL1 [*C. elegans*]	GGTTTAATTACCCAAGTTTGAG	
SL2 [*C. elegans*]	GGTTTTAACCCAGTTACTCAAG	

SL1a to SL1d are described in ref. [13]. The two canonical spliced leader sequences from *C. elegans *are listed for comparison. In cases where *A. avenae *ESTs are not listed, the respective cDNAs are incomplete at the 5' end and cannot be assessed for spliced leader use.

### Regulation of gene expression after exposure to environmental stress

The expression of 42 *A. avenae *genes in response to evaporative water loss (90% RH, 24 h), increased osmotic potential (1 M sucrose, 24 h), cold (4°C, 24 h) and heat (32°C, 24 h) was evaluated by quantitative PCR (Table 1; Table 2). Two thirds of the recognisable genes were found to be upregulated by drying, thus validating the selection procedure and implicating the EST set in anhydrobiosis. Interestingly, the large majority of genes analysed were also responsive to osmotic stress and cold (83% and 77%, respectively), probably reflecting similar effects on water activity and overlapping stress responses to all three environmental conditions. In contrast, heat stress caused relatively few genes to be induced, with 30% responding to elevated temperature, implicating a rather different response mechanism.

The degree of expression varied widely between genes although ANOVA analysis showed that, in most cases, a 1.5-fold increase in expression levels was significant (Table 1). Nevertheless, some genes were highly upregulated: the LEA protein and LEA-like genes (EF026241 and EF026240) showed a 43-fold and 36-fold increase in expression level, respectively, in response to desiccation, and a response to osmotic stress in excess of 20-fold. These genes were also substantially upregulated on exposure to low temperature (8-fold and 47-fold respectively). Other genes were not induced to the same levels in most cases, but notable results include that of a glutathione peroxidase (GR463914) whose mRNA concentration increased 32- and 14-fold in response to desiccation and osmotic stress, respectively; a DJ-1-like gene (GR463903) showed a 10-fold induction on desiccation; and an AHA1 activator gene (GR463897) also showed high relative expression levels following exposure to desiccation and osmotic stress. The above results are consistent with a requirement for increased antioxidant and molecular shield/chaperone activity during water stress, as has been noted previously (e.g. [4]). Of the novel genes (Table 2), four showed more than 10-fold upregulation on osmotic stress and two displayed over five-fold induction in response to desiccation.

### *A. avenae *is recalcitrant to RNA interference

Although the selective cloning procedure and the expression data of Tables 1 and 2 implicate the large majority of the gene set in anhydrobiosis, it would be desirable to obtain more direct evidence that they are required for survival of the dry state. To this end, we sought to establish RNA interference (RNAi) in *A. avenae *as a tool to selectively eliminate or reduce gene activity. Populations of *A. avenae *in which expression of particular genes had been reduced by gene silencing could then be assessed for desiccation tolerance; where survival was compromised, the respective genes would be strongly implicated in anhydrobiosis. RNAi occurs in *C. elegans *after injection of double-stranded RNA (dsRNA) corresponding to the target gene into worms [61], after soaking nematodes in a dsRNA solution [62] or after feeding nematodes on bacteria expressing dsRNA [63]. The last of these methods is not available to us since *A. avenae *do not feed on bacteria. Therefore, attempts were made at injecting dsRNA into *A. avenae *but these were unsuccessful due to technical difficulties relating to the small size of the worms. The injection method is also laborious and is not amenable to studies at the population level, which is usually required for assessment of desiccation tolerance. A soaking method was therefore attempted using dsRNA corresponding to the RNA polymerase II large (α) subunit gene, *ama-1*, which is known to give a lethal phenotype when silenced in *C. elegans *and other nematodes such as the human parasitic species *Brugia malayi *[64]. Two cDNA sequences corresponding to the *A. avenae ama-1 *transcript were cloned using primers directed against conserved peptide sequences identified in an alignment of nematode and other RNA polymerase II large subunit genes [65]. Soaking of *A. avenae *for 24 h in 1 mg/ml *ama-1 *dsRNA did not, however, show a phenotype compared to negative control experiments (data not shown). Positive technical controls were performed using *C. elegans *and cognate *ama-1 *sequences, resulting in sick animals within 24 h. Further controls were also carried out using *unc-22 *and GFP dsRNA on wild-type and GFP-expressing *C. elegans *(PD4251) to confirm the effectiveness of RNAi.

**Figure 2:**
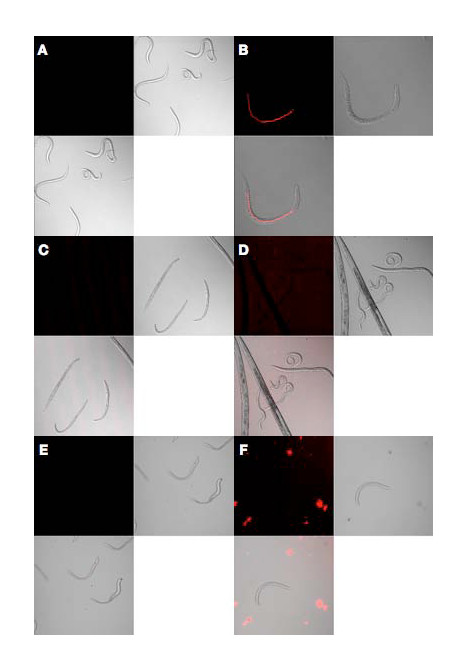
***A. avenae *dsRNA uptake assay**. *C. elegans *soaked in (A) unlabelled or (B) labelled dsRNA of GFP sequence. *A. avenae *soaked in (C, E) unlabelled or labelled (D, F) dsRNA of GFP sequence; in (E) and (F) 50 mM octopamine was also added.

To determine whether dsRNA molecules were taken up by *A. avenae*, nematodes were soaked in fluorescently-labeled dsRNA (GFP sequence) for a period of approximately 24 h. Wild type *C. elegans *was used as a positive control. Fluorescence was observed in the gut of *C. elegans *by confocal microscopy but no signal was observed in *A. avenae*, suggesting that dsRNA is not ingested efficiently in this species (Figure 2). Addition of the neuromodulator, octopamine, at concentrations up to 50 mM, which has been reported to improve dsRNA uptake in some parasitic nematodes [66,67], did not stimulate ingestion in *A. avenae*. Therefore, perhaps due to its feeding mechanism which involves stylet puncture of target fungal or plant tissues [68], it is likely that *A. avenae *is not readily amenable to RNAi by the soaking method.

### Cross-species RNAi of anhydrobiosis genes

One strategy which might partially circumvent difficulties with RNAi in *A. avenae *is cross-species gene silencing using *A. avenae *sequences to reduce expression of homologous transcripts in another anhydrobiotic nematode. Cross-species RNAi has been shown using sequences from the animal parasitic nematode, *Ascaris suum*, in *C. elegans*, for example [69]. Recent work by one of our groups (AMB) has demonstrated efficient systemic RNAi in *Panagrolaimus superbus*, a nematode in the same phylogenetic group as *A. avenae *with a marked resistance to desiccation. Indeed, since *P. superbus *is a bacterivore, it is susceptible to large scale RNAi in nematode populations using a feeding method very similar to that used routinely in *C. elegans *[70].

From the *A. avenae *EST set, 20 candidates (indicated in bold in Tables 1 and 2) were selected for RNAi in *P superbus*. The majority of these were chosen because they showed a high degree of similarity to sequences in *C. elegans *and/or *Caenorhabditis briggsae*, the rationale being that they were then more likely to have similar counterparts in *P. superbus*: both *P. superbus *and *A. avenae *are tylenchid nematodes, and thus more closely related in evolutionary terms than either is to the genus *Caenorhabditis *[70]. For comparison, three of the 20 ESTs chosen were novel sequences (GR463919, GR463921, GR463927).

*P. superbus *were fed bacteria expressing dsRNA corresponding to individual *A. avenae *sequences, after which worms were first pre-conditioned at 90% RH for 24 h followed by 24 h desiccation at 10% RH. The worms were then rehydrated overnight in buffer before scoring for survival. Worms fed dsRNA corresponding to GFP and treated similarly were taken as negative controls. Prior to desiccation, nematodes were also subjected to phenotypic analysis, but none of the experiments performed revealed any extraordinary change in morphology or behaviour, either of adults, larvae or embryos (Additional File 2).

**Figure 3:**
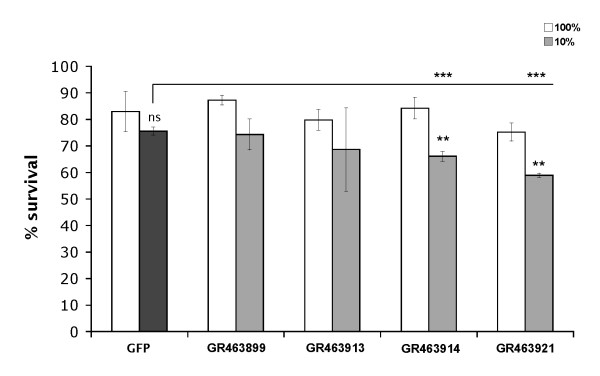
**Cross-species RNAi using *A. avenae *EST sequences to induce gene silencing in *P. superbus***. *P. superbus *larvae were subjected to RNAi for 11-13 days with selected *A. avenae *genes (GR463899, *ggr-3*; GR463913, *col-14*; GR463914, glutathione peroxidase; and GR463921, novel) identified in a preliminary screen. GFP was taken as a negative control for RNAi. The resulting mixed stage worms were subjected to desiccation at 10% RH for 24 h (negative control - black bar; other samples - grey bars). All samples were also subjected to mock desiccation at 100% RH (white bars). Mean values of triplicates, together with standard deviations, are shown; a one-way ANOVA with Tukey post-hoc test was performed on GR463914 and GR463921: *, significance at P < 0.05; **, significance at P < 0.01; ***, significance at P < 0.001; ns, not significant.

This first drying experiment was used as a preliminary screen; four candidates which showed a reduction in desiccation tolerance of at least 10% compared to controls were chosen for a second round of RNAi and desiccation: GR463899 (*ggr-3*, a predicted member of the GABA family of ligand-gated chloride channels); GR463913 (*col-14*, a collagen structural gene); GR463914 (a glutathione peroxidase); and GR463921 (unknown function). Survival of *P. superbus *after RNAi treatment and desiccation was compared to nematode viability after mock desiccation, in which worms were subjected to 100% RH; as previously, a GFP negative control was also performed (Figure 3). In this experiment, RNAi treatment with both GR463899 and GR463913 resulted in no significant reduction in survival following desiccation compared to non-dried and GFP controls. Therefore, neither was considered to have a measurable RNAi effect. However, GR463914 and GR463921 did show significant (P < 0.01) reduction in survival of drying after RNAi to 66% and 59%, compared to 84% and 75%, respectively, without desiccation. These results therefore suggest that glutathione peroxidase and an additional uncharacterised gene (GR463921) are contributors to desiccation tolerance in *P. superbus*, and by extension in *A. avenae*.

### Glutathione peroxidase gene activity contributes to anhydrobiosis in *P. superbus*

If the cross-species strategy used above is valid, then genes that are upregulated in response to desiccation in *A. avenae*, and that have well-conserved sequence counterparts in *P. superbus*, should be part of an anhydrobiotic gene set in both nematodes. Therefore, we attempted to identify genes in *P. superbus *which were homologues or paralogues of the two genes indicated by the cross-species RNAi experiments, in order to carry out similar experiments with cognate *P. superbus *sequences. Consequently, an EST library of ~9,000 *P. superbus *cDNAs (Tyson *et al*., in preparation) was searched for sequences corresponding to *A. avenae *GR463914 and GR463921. Although no matches were found for the novel sequence represented by GR463921, two different sequences showing similarity to GR463914 were obtained, Ps92 (accession number GR881192) and Ps114 (accession number GR881191), both of which are similar to glutathione peroxidase sequences in GenBank and other databases. For example, both Ps92 and Ps114 gave highly significant matches with the *C. elegans *glutathione peroxidase gene C11E4.1, showing BLASTX scores of 2E-97 and 1E-86, respectively. The ESTs probably represent different genes in *P. superbus *since they are only 66% identical using the ALIGN tool.

**Figure 4:**
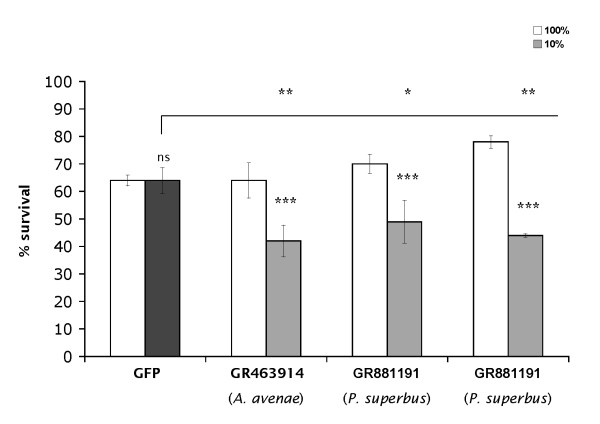
**RNAi of cognate glutathione peroxidase transcripts in *P. superbus *reduces desiccation tolerance**. Feeding of *P. superbus *with bacteria expressing dsRNA corresponding to *A. avenae *glutathione peroxidase sequence, GR463914, and *P. superbus *glutathione peroxidase sequences, GR881191 and GR881192, followed by assessment of desiccation tolerance. A bacterial feeder strain expressing dsRNA corresponding to GFP was used as a negative control. Mixed stage worms were subjected to desiccation at 10% RH for 24 h (negative control - black bar; other samples - grey bars). All samples were also subjected to mock desiccation at 100% RH (white bars). Mean values of triplicates, together with standard deviations, are shown; a one-way ANOVA with Tukey post-hoc test was performed: *, significance at P < 0.05; **, significance at P < 0.01; ***, significance at P < 0.001; ns, not significant.

RNAi experiments were therefore carried out in *P. superbus *with these sequences, together with *A. avenae *GR463914 (glutathione peroxidase) and GFP sequences as positive and negative controls, respectively. Figure 4 confirms that RNAi using the *A. avenae *glutathione peroxidase sequence reduces desiccation tolerance in *P. superbus*. Moreover, the two glutathione peroxidase sequences from the *P. superbus *EST library also showed a pronounced RNAi effect on survival after drying. These results strongly implicate a role for glutathione peroxidases in nematode anhydrobiosis and provide the first genetic evidence for the importance of antioxidants in metazoan desiccation tolerance.

## Discussion

Anhydrobiosis is one of the most fascinating biological phenomena, yet the molecular mechanisms involved are still relatively poorly understood. The application of recently-developed molecular techniques to the study of anhydrobiosis has been hampered by the lack of a suitable desiccation-tolerant model organism, certainly among the metazoans, for which genome sequence and genetic tools are not yet available. Perhaps the best-characterised metazoan example is the nematode, *A. avenae*, in which the physiology and biochemistry of anhydrobiosis has been studied since the 1970s (e.g. [6]). To gain insight into the set of genes employed by *A. avenae *to achieve the anhydrobiotic state, which can be termed the anhydrobiotic gene set, or the desiccome [71], we have used a selective cloning technique to identify genes that are upregulated by evaporative water loss. The majority of genes identified were confirmed as desiccation-inducible using real-time qPCR, thus validating the cloning procedure; many examples of this gene set were also found to be responsive to osmotic stress and reduced temperature, possibly due to a common effect on water activity in all three stress conditions. Relatively few members of the EST set were responsive to elevated temperature, however. This fits with general observations in other invertebrates and plants where a large overlap in the stress responses to dehydration, osmotic stress and cold is observed, but where heat stress activates a different response pathway [72]. The complexity of the response to water loss is illustrated by the involvement of molecular chaperone/shield proteins, redox balancing systems, osmolyte biosynthetic enzymes, C-type lectins, protein and polysaccharide metabolic enzymes, as well as genes controlling gene transcription and signal transduction pathways. In particular, new LEA and LEA-like protein genes were identified suggesting that several proteins of this type are involved in the response to water stress in invertebrates, as in plants. It seems unlikely, however, that the diversity of the LEA proteome in invertebrates will rival that of plants, where >50 LEA protein genes can be found in a single organism [73,74]; in *C. elegans*, for example, only three such genes have been noted [24].

While such information on transcriptional stress responses can be extremely useful in assessing which genes and regulatory systems are involved in combating the stress imposed, it is becoming clear, at least in some organisms such as yeast, that not all stress-induced genes are essential for stress tolerance. Thus, in *Saccharomyces cerevisiae*, for example, very few genes required for DNA repair were induced by DNA-damaging agents [75]. Other researchers report similar conclusions for yeast subjected to osmotic stress [76], oxidative stress [77], and anaerobic growth conditions [78]. It is therefore important, if possible, to go beyond a purely correlative study, where stress-induced genes are catalogued, to experiments where genes of interest can be mutated or silenced and the effect of this on stress tolerance determined.

Silencing of specific gene targets by RNAi has been invaluable in nematodes, in particular *C. elegans*, as an alternative to mutagenesis that allows gene function to be probed on a large scale [79,80]. Such high throughput functional genomics would be ideal for identifying the anhydrobiotic gene set in a nematode such as *A. avenae*. Unfortunately, attempts to perform RNAi on *A. avenae *by microinjection, or by soaking in dsRNA corresponding to the gene target, were not successful. This might be due to resistance or partial resistance to RNAi in this species, as has been found in some animal parasitic nematodes [81]. However, with the soaking method, recalcitrance might simply reflect poor ingestion of surrounding medium by *A. avenae*, since fluorescently labelled dsRNA was not visible in its intestine. Alternative RNAi strategies might be to attempt a biolistic delivery method, as recently demonstrated in dehydration-stressed barley leaves [82], or to introduce dsRNA by electroporation, as described for the gastrointestinal parasite *Trichostrongylus colubriformis *[83]. However, such RNAi techniques would be difficult to adapt for a high throughput screening experiment.

The cross-species method developed here, which exploits the relative ease with which RNAi can be performed in *P. superbus *[70], was extremely useful in allowing a limited screen of *A. avenae *sequences. Only two out of 20 *A. avenae *ESTs tested gave a marked reduction in *P. superbus *desiccation tolerance, but this probably reflects a requirement by the RNAi machinery for precise sequence matching between the short interfering RNAs generated from dsRNA and the target mRNA [84]; sequence divergence of homologous RNA targets will reduce the efficacy of RNAi across species. It is also formally possible that, as in yeast, many genes induced by water stress in *A. avenae *are not required for stress tolerance in *P. superbus*, or indeed in *A. avenae*. Nevertheless, the observation that silencing of two particular transcripts reduces survival of drying in *P. superbus *is the first such demonstration in any desiccation-tolerant organism and demonstrates the potential for RNAi in dissecting anhydrobiosis and determining the desiccome. This result also demonstrates the advantage of *P. superbus *as a model anhydrobiote. Accordingly, a large cognate EST dataset has already been assembled for high throughput screening by RNAi for genes required in anhydrobiosis in this nematode; a number of interesting candidates have been identified and are currently being characterised (Tyson *et al*., unpublished). The availability of a panel of other *Panagrolaimus *species of varying degrees of desiccation tolerance will also be useful for comparative studies of the phenomenon [7].

Of the two *A. avenae *ESTs that gave a phenotype in the cross-species RNAi desiccation experiments, one was a novel sequence not represented in sequence collections. This EST, GR463921, is only 350 bp in length and is not full length, since it lacks clearly defined 5' and 3' ends, as would be signified by a spliced leader and polyA tail, respectively (although a proportion of nematode mRNAs lack spliced leaders; [59]). Nevertheless, it shows modest upregulation during desiccation and osmotic stress, and merits further investigation by isolation of full length cDNA clones from *A. avenae *and a search for cognate sequences in *P. superbus*; the latter must exist for the cross-species RNAi to have been successful. However, an alternative role for GR463921 might be as a non-coding RNA involved in regulating anhydrobiosis in nematodes. A precedent for this exists in the desiccation-tolerant resurrection plant, *C. plantagineum*, where an abundant, small non-coding RNA (CDT-1) is able to induce tolerance in callus tissue [85]. CDT-1 is a member of a family of retroelements which govern synthesis of siRNA involved in control of desiccation tolerance pathways [86]. Formation of dsRNA is probably important for CDT-1 function and, intriguingly, the GR463921 sequence is predicted to form a high degree of secondary structure (Additional File 3) by the RNAfold algorithm ([87]; http://rna.tbi.univie.ac.at). It will therefore be of interest to investigate this transcript further with a view to understanding its precise role in nematode anhydrobiosis.

The second EST giving an RNAi phenotype on desiccation, which matches glutathione peroxidase sequences in GenBank and other sequence collections, was a full length cDNA of 649 bp including its spliced leader (SL1a) but excluding its polyA tail. It appears to contain an open reading frame of 151 amino acids (Mr 17480) bounded by a UGA stop codon. However, some glutathione peroxidases are known to contain selenocysteine [88,89] which is not directly represented in the genetic code, but is incorporated into nascent protein when the ribosome reads UGA in the presence of a selenocysteine insertion element in the mRNA [90]. Therefore, the predicted open reading frame might be shorter than the actual protein length; indeed, there are two in-frame UGA codons before a third, TAA, stop codon is encountered, consistent with up to two selenocysteines being incorporated into the *A. avenae *glutathione peroxidase. The two *P. superbus *glutathione peroxidase ESTs, GR881191 (Ps114) and GR881192 (Ps92), are somewhat longer than the *A. avenae *example, at 735 bp and 808 bp, respectively. Ps114 does not quite extend to the 3' end, since its open reading frame of 230 amino acids lacks a stop codon, while the reading frame of Ps92 is likely complete at 228 residues. Interestingly, both predicted proteins from *P. superbus *have a hydrophobic sequence of about 20 amino acids at their N-terminal ends consistent with an endoplasmic reticulum translocation signal. Both proteins are therefore probably secreted; indeed, the best BLAST matches are with cuticular glutathione peroxidases from other nematodes. For example, the Ps114 protein gives a score of 7E-70 with the cuticular glutathione peroxidase from *Brugia pahangi*, also known as its major surface antigen gp29. In contrast, the *A. avenae *protein does not contain a signal peptide and therefore is probably intracellular. As such, the *A. avenae *and *P. superbus *glutathione peroxidases described are likely to have different locations within each nematode, and to have different substrate preferences, with the secreted *P. superbus *examples not metabolising hydrogen peroxide but instead larger hydroperoxide substrates such as lipid peroxides. The fact that both types of enzyme are implicated in anhydrobiosis is consistent with the need to protect both intracellular and membrane targets from oxidative stress during desiccation.

To further investigate the role of antioxidants in nematode anhydrobiosis, we also identified a third *P. superbus *EST, Ps41 (accession number GR881190), which is likely to encode a peroxiredoxin (also called thioredoxin peroxidase) since it gave a highly significant BLASTX match (6E-47) with the *C. elegans prdx-6 *gene. Like glutathione peroxidases, peroxiredoxins are also thiol peroxidases involved in the control of cellular peroxide levels, and both classes of enzyme are evolutionarily related [91]. Preliminary, unpublished results suggest that RNAi treatment with peroxiredoxin dsRNA also reduces survival of desiccation in *P. superbus*, consistent with a role for a second type of thiol peroxidase in nematode anhydrobiosis.

Oxidative stress is experienced by organisms undergoing a wide range of abiotic stress conditions, probably due to malfunction of mitochondria (and chloroplasts in plants) resulting in generation of reactive oxygen species [92]. Accordingly, an oxidative stress response forms part of the environmental stress response defined by Gasch et al. [93] in yeast and is part of the minimal stress response described by Kültz [94]. Desiccation stress is no exception and it has long been recognised that antioxidants are likely to play a role in desiccation tolerance (reviewed in [95-97]). Recent global studies in plants have reinforced this perception (reviewed in [98]) and, for example, peroxiredoxin and glutathione peroxidase, amongst other antioxidants, are induced by dehydration in desiccation-tolerant resurrection plants [99,100].

The demonstration of a role for glutathione peroxidase in nematode anhydrobiosis is consistent with, and validates, the many correlative studies which have previously implicated antioxidants. In nematodes, for instance, three studies have identified dehydration-induced genes encoding antioxidant enzymes: Gal *et al*. [8] described a number of ESTs from *S. feltiae*, a desiccation-resistant entomopathogenic species, including a glutathione peroxidase; in *A. avenae*, a glutaredoxin gene was induced by drying, together with an LEA protein gene and a gene encoding another hydrophilic protein, anhydrin [24]; and, in a recent large-scale study of the Antarctic nematode *P. murrayi *by Adhikari *et al*. ([25]; and see review by Wharton and Marshall [101]), genes encoding glutathione-S-transferase, superoxide dismutase, peroxiredoxin and glutathione peroxidase were included in a dehydration-induced EST panel. The present study takes our understanding of anhydrobiosis a step further by showing a reduced level of desiccation tolerance after silencing glutathione peroxidase transcripts, thus clearly demonstrating a stress-combative role for antioxidants. Further work will explore gene silencing more extensively in *P. superbus *to determine the desiccome in this species in more detail.

## Conclusions

Using a combination of differential cloning, expression profiling and cross-species RNAi, we have identified a panel of genes implicated in the response to desiccation in the anhydrobiotic nematode, *A. avenae*. The gene set includes novel hydrophilic proteins, one of which is a previously-undescribed LEA protein, and other stress response genes controlling synthesis of compatible solutes, chaperone systems and antioxidants. The large majority of these genes were upregulated by evaporative water loss, and many also by osmotic upshift and low temperature, conditions which also involve water stress. We also presented evidence for RNAi recalcitrance in *A. avenae*, but were able to show cross-species RNAi of two *A. avenae *sequences in another anhydrobiotic nematode, *P. superbus*. One of these was a novel sequence, which is potentially a non-coding regulatory RNA, and the second encoded an intracellular glutathione peroxidase: both reduced survival of desiccation of *P. superbus *after RNAi treatment. Two cognate glutathione peroxidase sequences from *P. superbus *were also identified and, when used for RNAi, were shown to have a similar effect on desiccation tolerance. These results therefore demonstrate the complex nature of desiccation tolerance and emphasise the need for effective antioxidant systems in anhydrobiosis.

## Methods

### Culture, desiccation and stress treatment of nematodes

*Aphelenchus avenae *was grown in the dark at 20°C on the fungus *Rhizoctonia solani*, itself grown on a substrate of wheat [102]. After a growth period of 2-3 weeks (before swarming of the nematodes in the jars), nematodes were rinsed with tap water and brought to a concentration of approximately 3,300 worms per ml. Nematodes were cleaned in 30% (w/v) sucrose solution, followed by three washes in tap water and the nematodes were transferred to 25 mm Supor^® ^Membrane Disc Filters (0.45 μm, Pall Corporation, Michigan, USA; 30,000 nematodes per filter) by vacuum filtration. These were placed in a desiccation chamber at 20°C for 24 h over a saturated solution of MgSO_4_.7H_2_O (90% RH), or over a saturated solution of KCl (85% RH) [103].

For the qPCR experiments the nematodes were grown and harvested as described above but they were cleaned by placing the nematode pellet in a sieve containing a layer of Kleenex^® ^allowing them to crawl through the paper (for 2-3 h) into the water filled dish in which the sieve had been placed. *A. avenae *populations were exposed to individual stresses as follows. Heat stress: one replicate consisted of 1 ml of the above worm suspension placed in a 30 mm Petri dish incubated at 32°C for 24 h. Cold stress: one replicate consisted of 1 ml of worm suspension in a 30 mm Petri dish incubated at 4°C for 24 h. Desiccation: one replicate consisted of 1 ml of worm suspension vacuum filtered onto a 25 mm Supor^® ^membrane disc and placed in a desiccation chamber set at 90% RH (using a saturated solution of MgSO_4_.7H_2_O) for 24 h. Osmotic stress: one replicate consisted of 1 ml of worm suspension added to 1 ml of 1 M sucrose solution in a 30 mm Petri dish and placed at 20°C for 24 h. Control nematodes consisted of 1 ml of worm suspension in 30 mm Petri dishes incubated at 20°C for 24 h. Three biological replicates were set up for each stress and for control worms. These stress conditions yielded an 80% survival rate for each stress compared to a 90% survival rate for the control worms.

*P. superbus *and *C. elegans *were grown on NGM plates and fed *Escherichia coli *strain OP50, or strain HT115 carrying an empty L4440 plasmid, in the dark at 25°C.

### Construction of a desiccation induced EST library

Total RNA was extracted from desiccated and control nematodes using TRI^® ^Reagent (Sigma-Aldrich GmbH, Steinheim, Germany). Total RNA from desiccated nematodes was converted to 5'-oligo-capped cDNA using the GeneRacer™ Kit (Invitrogen, Carlsbad, California, USA), following the manufacturer's instructions. The GeneRacer™ Oligo dT primer was used in the reverse transcription reaction. PCR was carried out on the 5'-oligo-capped cDNA with the GeneRacer™ 5'-primer (CGACTGGAGCACGAGGACACTGA) and the GeneRacer™ 3'-primer (GCTGTCAACGATACGCTACGTAACG) using Platinum^® ^*Taq *DNA Polymerase (Invitrogen). The product of this reaction was cloned into the pCR2.1 TOPO vector using a TOPO-TA Cloning kit (Invitrogen).

### Reverse Northern hybridization

Recombinant clones were PCR amplified in a 96 well plate using standard M13 forward and reverse primers and the presence of an insert was confirmed by electrophoresis on a 1% agarose gel. The PCR products were denatured by adding NaOH to a total concentration of 0.4 M and EDTA to a total concentration of 0.04 M and heating at 99°C for 10 min. The denatured DNA was blotted onto Hybond™-N+ transfer membrane (Amersham Biosciences, Buckinghamshire, UK) in duplicate using a SRC 60-D Dot Blot Minifold (Schleicher & Schuell, Dassel, Germany). The membranes were air dried at 55°C for 30 min and UV-crosslinked using a UV Stratalinker^® ^1800 (1200 mJ; Stratagene, California, USA).

### Screening the EST library

Total RNA was extracted from desiccated (90% RH for 24 h) and control nematodes as before [24]. The RNA was resuspended in 30 μl DEPC-treated H_2_O, concentration determined by measuring absorbance at 260 nm and adjusted to 1 μg/μl. 5 μg of RNA was converted to cDNA using the SuperScript™ First-Strand Synthesis System (Invitrogen). Experimental (desiccated) and control probes were constructed by radiolabelling this cDNA with [^32^P]-dCTP using the Prime-a-Gene^® ^Labelling System (Promega, Southampton, UK) in accordance with the manufacturer's instructions. One replicate of each dot blot membrane was hybridized with the experimental probe and the corresponding replicate was hybridized with the control probe. Hybridisation was carried out in hybridisation buffer (5% dextran sulfate, 1 M NaCl, 1% SDS in water) at 60°C for 18 h in glass tubes with gentle rotation. The membranes were washed twice in wash buffer (10× SSC, 0.5% SDS in water) for 4 min at room temperature followed by two washes for 15 min at 60°C. The membranes were then exposed on X-OMAT AR photography paper (Kodak, New York, USA) for 24 h. The control and experimental dot blots were digitally scanned and analyzed using the ImageQuant TL Array Analysis software (Amersham Biosciences). Clones considered to be upregulated in response to desiccation were selected for sequencing and sent to Agowa GmbH (Berlin, Germany). The resulting sequences were assembled into contigs using the online sequence assembly program CAP3 http://pbil.univ-lyon1.fr/cap3.php, yielding a dataset of 88 unique ESTs, enriched in those upregulated in response to desiccation.

### Bioinformatics

The POPPs [35] is a suite of applications, one of which clusters protein sequences based on similarities in their peptide compositions, in this case to see whether the input sequence clusters with any of the LEA protein groups and, if so, which. The GRAVY calculation was undertaken by a small Python application which uses the Eisenberg Consensus scale [104] in preference to the Kyte-Doolittle scale [105], which has well known anomalies. Three predictors of unfolded proteins were used in this study: Foldindex ([106]; http://bip.weizmann.ac.il/fldbin/findex/, VL-XT ([107]; http://www.pondr.com) and IUPred ([108]; http://iupred.enzim.hu). Uversky plots [109] of charge vs. hydropathy were performed at pondr.com and raw data downloaded to construct graphical output in GraphPad Prism version 4.0b (GraphPad Software, California, USA). An application called Perdix was used to search the input sequences for amino acid periodicity, i.e. amino acids that recur every N amino acids. So, for example, in the peptide GAGPG the G recurs with period 2. The application Perdix, and the earlier application for computing hydrophobicity and other peptide statistics, both currently unpublished, can be obtained from Wise. The pairwise sequence alignment application ALIGN [110] is part of the San Diego Supercomputer Workbench http://workbench.sdsc.edu.

### Quantitative real time PCR

The replicate worm samples from each individual stress treatment were pooled and RNA was extracted using Trizol reagent for each stressed and control worms followed by treatment with DNAse I (RNase free, Invitrogen). The concentration and quality of RNA was determined using a Thermo Scientific Nanodrop Spectrophotometer. Total RNA (1 μg per reaction) was converted to cDNA using the Roche Transcriptor First Strand cDNA Synthesis Kit. Primers were designed for each gene to amplify a fragment of approximately 125 bp (see Additional File 4). Real time qPCR reactions were carried out with a Roche LightCycler 480 thermocycler using Roche SYBR I Master Mix with 0.0025 pmol primers (each), 500 ng of sample cDNA template and 0.5 U heat-labile uracil-N-glycosolase per reaction. Relative expressions were calculated using the second derivative maximum method with *A. avenae *5.8S rRNA and *ama-1 *genes as reference genes. Statistically significant differences in expression levels were confirmed using ANOVA with a confidence level of 99.9% and Dunnett's comparison test with α = 1.

### Assessing *A. avenae *for RNAi capability

Two *ama-1 *cDNA fragments were obtained from *A. avenae *by RT-PCR. The short fragment (322 bp), was obtained with the primers AaAMA1s (5'-TTTTTCATGCAATGGGCGGTC-3'; forward) and AaAMA1as (5'-ACATAAACCTCTCTCACGACG-3'; reverse). Reverse transcription was performed using SuperScript II (Invitrogen) according to manufacturer's instructions, and the PCR was done under the following conditions: 95°C 5 min; then 35 cycles of 95 C 30 s, 57°C 30 s and 72°C 1 min; with a final extension at 72°C for 10 min. A longer version of *ama-1 *cDNA (800 bp) was cloned using the same RT-PCR conditions with primers Aa.AMA1.s2 (5'-ACGTCGACTGATTAAGGCCAT-3'; forward) and Aa.AMA1.as3 (5'-TGATGATCTCCTTCAGACGAG-3'; reverse). PCR products underwent a second amplification round using T7-tagged primers. These new amplicons were then used for *in vitro *transcription using Ampliscribe T7 Flash Transcription Kit (Epicentre) according to manufacturer's instructions. For RNAi attempts, worms were soaked in 20 μl soaking buffer (5 mM Tris-HCl pH7.0) containing dsRNA (1 μg/μl) for 24 h and then plated on LB agar plates (50 μg/ml carbenicillin). Worms were placed over a small spot of fungus (*Botrytis cinerea*) previously grown on the LB agar plate for 24 h. Survival analysis was performed by observation under a microscope. *C. elegans *was treated in a similar fashion except worms were incubated with GFP and *unc-22 *dsRNA for 24 h at ambient temperature and recovered on *E. coli*-seeded NGM plates until phenotypes were observed. For dsRNA uptake analysis, dsRNA molecules were first synthesised by *in vitro *transcription from undigested L4440-GFP plasmids. Then 5 μg dsRNA was labelled using Label IT CX-rhodamine Nucleic Acid Labeling Kit according to manufacturer's instructions (Mirus/Cambridge Bioscience). Worms were soaked in 20 μl soaking buffer for 22 h at 15°C in the dark. Uptake efficiency was assessed using a confocal microscope (Zeiss LSM510 META).

### Cross-species RNA interference and desiccation tolerance

Plasmid DNA of selected *A. avenae *EST clones (in TOPO vector; Invitrogen) were digested using *Sac *I (NEB) and *Xho *I (NEB), inserts were gel-purified and ligated into *Sac *I/*Xho *I -digested L4440 feeding vector [80]. Competent HT115 cells were transformed with ligation products and selected on LB ampicillin (100 μg/mL). Subcloning was confirmed by digesting L4440 plasmids with *Sac *I/*Xho *I. Transformed HT115 clones containing individual plasmids for RNAi were each grown in 10 ml LB ampicillin (50 μg/mL) overnight, recovered by centrifugation and resuspended in 300 μl plain LB medium. Bacteria were transferred to Petri dish plates (9 cm diameter) containing NGM agar plus IPTG (240 μg/ml) and ampicillin (50 μg/ml). Plates were left for 24 h at ambient temperature to allow dsRNA induction. *P. superbus *was prepared for RNAi as follows: a large (140 mm) NGM plate containing mixed stage animals with many gravid adults was harvested, worms washed 3× in M9 buffer and synchronised by bleaching (3 min in 1% NaOCl, 0.5 M NaOH). After bleaching, worms were washed 3× in M9 and left to hatch in M9 on a shaker for 48 h. Approximately 1500 worms were placed onto each feeding plate (three per test gene) where they remained for approximately 12 days in the dark at 25°C before desiccation. Worms were then collected, washed 3× in M9 buffer, counted and placed onto three 25 mm Supor^® ^Membrane Disc Filters by vacuum filtration (approximately 300 RNAi-treated worms per filter) and processed as follows: 24 h at 90% RH (saturated BaCl_2_); 24 h at 10% RH (dry silica gel) and overnight in M9 buffer. Non-desiccated control worms were processed as follows: 48 h at 100% RH (UHP H_2_O) and 24 h in M9 buffer. All treatments were in 350 ml sealed plastic boxes. After rehydration, viability was assessed under the microscope, where 80 worms were randomly chosen for analysis from each filter (spontaneously moving worms were considered alive). *C. elegans *fed with a clone expressing *unc-22 *dsRNA was used as a positive control of RNAi treatment (twitching phenotype). Negative controls consisted of *P. superbus *and *C. elegans *worms fed with a clone expressing GFP (green fluorescent protein) dsRNA. Statistical relevance was determined by one-way ANOVA and a Tukey post hoc test using InStat3 (GraphPad Software, San Diego, CA).

## Authors' contributions

WR and KMD prepared and screened the *A. avenae *cDNA libraries. WR sequenced and characterized the selected differentially expressed cDNA sequences. TT performed the qPCR experiments and provided material for the manuscript. BAC grew *A. avenae*, carried out nematode stress treatments and prepared cDNA. AMB did the phylogenetic reconstruction. SC, TCP and MCB designed, performed and interpreted RNAi experiments, and provided material for the manuscript. MJW performed bioinformatics and provided material for the manuscript. AMB and AT analysed sequence data, and designed and coordinated the study. WR, AMB and AT drafted the manuscript. All authors read and approved the final manuscript.

## Supplementary Material

Additional file 1 The molecular phylogeny of representative polygalacturonase protein sequences from bacteria, fungi and plants. The phylogenetic positions of the nematode and insect polygalacturonase sequences, postulated to have been acquired by horizontal gene transfer, are indicated in blue. Sequence alignments were constructed using Clustal W^1 ^and the unrooted phylogenetic tree was constructed using the Neighbor-Joining Method^2 ^as implemented by MEGA (version 3.1)^3^. All sites containing alignment gaps and missing information were removed from the analysis. The Poisson correction distance for multiple substitutions at the same site was used and substitution rates among sites were considered to be different (the gamma shape parameter was set at α = 2). The accession numbers of the polygalacturonase sequences are as follows: *Aphelenchus avenae*, GR463895; *Aphelencus avenae*, GR463896; *Arabidopsis thaliana*, AAL32525; *Aspergillus awamori*,BAA95407; *Chrysomela tremulae*, ACP188314; *Clostridium acetobutylicum*, NP_350265; *Colletotrichum lupini*, ABL01533; *Erwinia chrysanthemi*, CAB99319; *Fusarium oxysporum*, BAE97103; *Glycine max*, AAD46484; *Medicago sativa*, CAA72003; *Klebsiella oxytoca*, AAL49975; *Meloidogyne incognita*,AAM282405; *Neosartorya fischeri*, XP_001266657; *Penicillium chrysogenum*, CAP99317; *Penicillium griseoroseum*,AAF03895; *Ralstonia solanacearum*, YP_002253951; *Sitophilus oryzae*, AAG35693.1; *Sorghum bicolor*, XP_002455500; *Treponema pectinovorum*, AAT11785; *Vitis vinifera*, ABW76153; *Yersinia intermedia*; ZP_04637902; *Zea mays*, NP_001150436.
To access the proteins via the NCBI protein database please search them via the following link: http://www.ncbi.nlm.nih.gov/protein/. Click here for file

Additional file 2 Phenotypic analyses after RNAi treatment with ESTs listed. Click here for file

Additional file 3 Predicted secondary structure of RNA represented by EST GR463921 using RNAfold at http://rna.tbi.univie.ac.at with default settings. The colour scale indicates likelihood of structure formation. Click here for file

Additional file 4 Primer sequences used for quantitative real-time PCR of ESTs (see Tables 1 and 2). Click here for file
