# Supplementary material for: Expression profiling and cross-species RNA interference (RNAi) of desiccation-induced transcripts in the anhydrobiotic nematode Aphelenchus avenae
Source: BMC Mol Biol. 2010 Jan 19;11:6. doi: 10.1186/1471-2199-11-6 (PMC2825203; doi:10.1186/1471-2199-11-6)
Supplement: Additional file 1 — The molecular phylogeny of representative polygalacturonase protein sequences from bacteria, fungi and plants. The phylogenetic positions of the nematode and insect polygalacturonase sequences, postulated to have been acquired by horizontal gene transfer, are indicated in blue. Sequence alignments were constructed using Clustal W1 and the unrooted phylogenetic tree was constructed using the Neighbor-Joining Method2 as implemented by MEGA (version 3.1)3. All sites containing alignment gaps and missing information were removed from the analysis. The Poisson correction distance for multiple substitutions at the same site was used and substitution rates among sites were considered to be different (the gamma shape parameter was set at α = 2). The accession numbers of the polygalacturonase sequences are as follows: Aphelenchus avenae, GR463895; Aphelencus avenae, GR463896; Arabidopsis thaliana, AAL32525; Aspergillus awamori,BAA95407; Chrysomela tremulae, ACP188314; Clostridium acetobutylicum, NP_350265; Colletotrichum lupini, ABL01533; Erwinia chrysanthemi, CAB99319; Fusarium oxysporum, BAE97103; Glycine max, AAD46484; Medicago sativa, CAA72003; Klebsiella oxytoca, AAL49975; Meloidogyne incognita,AAM282405; Neosartorya fischeri, XP_001266657; Penicillium chrysogenum, CAP99317; Penicillium griseoroseum,AAF03895; Ralstonia solanacearum, YP_002253951; Sitophilus oryzae, AAG35693.1; Sorghum bicolor, XP_002455500; Treponema pectinovorum, AAT11785; Vitis vinifera, ABW76153; Yersinia intermedia; ZP_04637902; Zea mays, NP_001150436. To access the proteins via the NCBI protein database please search them via the following link: http://www.ncbi.nlm.nih.gov/protein/. [file 1471-2199-11-6-S1.PDF]

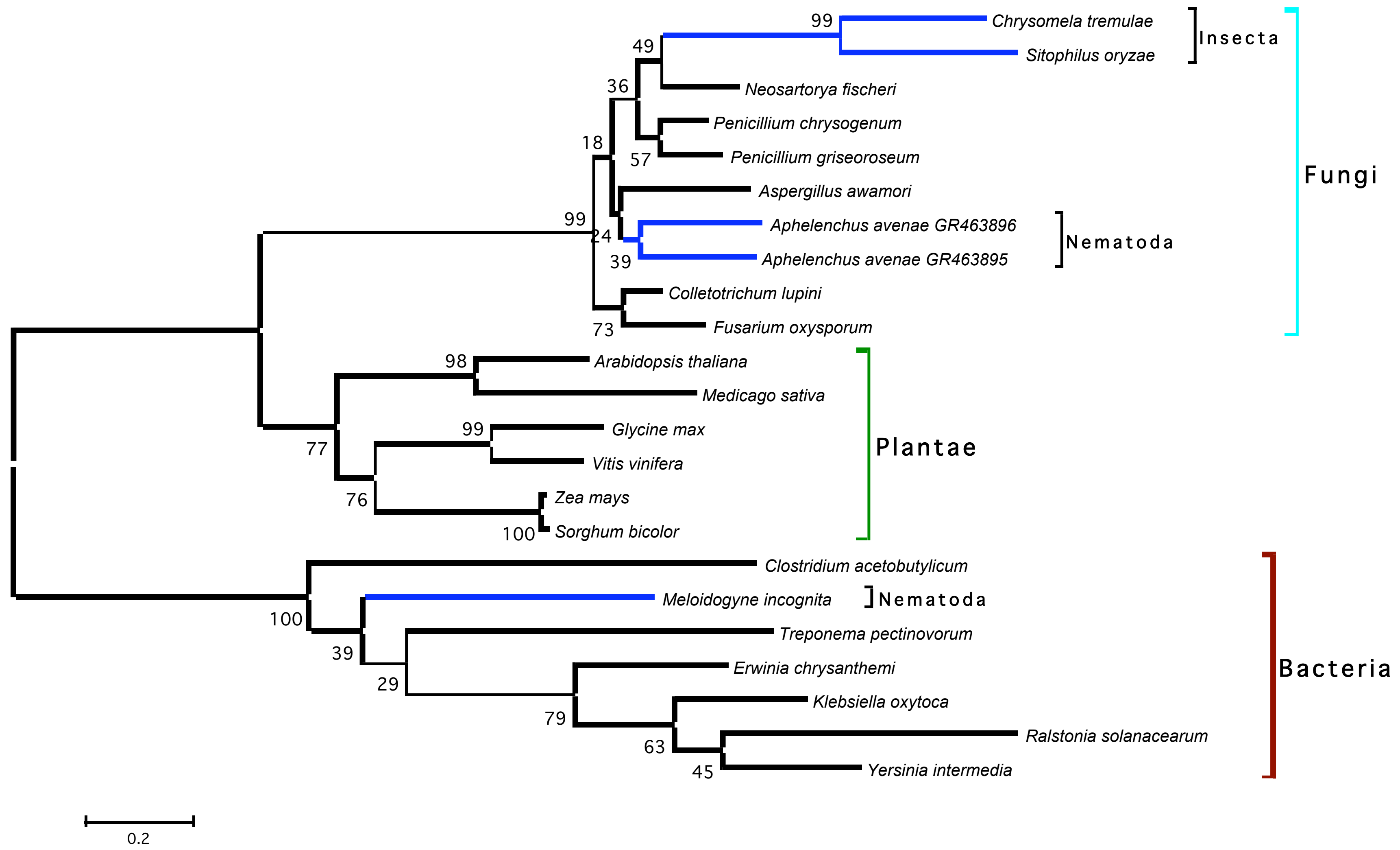

Chrysomela tremulae
Sitophilus oryzae
Insecta
Neosartorya fischeri
Penicillium chrysogenum
Penicillium griseoroseum
Aspergillus awamori
Aphelenchus avenae GR463896
Aphelenchus avenae GR463895
Nematoda
Colletotrichum lupini
Fusarium oxysporum
Fungi
Arabidopsis thaliana
Medicago sativa
Glycine max
Vitis vinifera
Zea mays
Sorghum bicolor
Plantae
Clostridium acetobutylicum
Meloidogyne incognita
Nematoda
Treponema pectinovorum
Erwinia chrysanthemi
Klebsiella oxytoca
Ralstonia solanacearum
Yersinia intermedia
Bacteria
99
49
36
18
57
99
24
39
73
98
77
99
76
100
100
39
29
79
63
45
0.2

References

1. Thompson JD, Higgins DG, Gibson TJ: **Clustal W - improving the sensitivity of progressive multiple sequence alignment through sequence weighting, position-specific gap penalties and weight matrix choice.** *Nucl Acids Res* 1994, **22:**4673-4680.
2. Saitou N, Nei M: **The Neighbor-Joining method - a new method for reconstructing phylogenetic trees.** *Mol Biol Evol* 1987, **4:**406-425.
3. Kumar S, Tamura K, Nei M: MEGA3: **Integrated software for molecular evolutionary genetics analysis and sequence alignment.** *Brief Bioinformat* 2004, **5:**150-163.
4. Pauchet Y et al.: **Pyrosequencing of the midgut transcriptome of the poplar leaf beetle *Chrysomela tremulae* reveals new gene families in Coleoptera.** *Insect Biochem Mol Biol* 2009, **39:**403-413.
5. Jaubert S, Laffaire JB, Abad P, Rosso MN: **A polygalacturonase of animal origin isolated from the root-knot nematode *Meloidogyne incognita*.** *FEBS Lett* 2002, **522:**109-112.
6. Shen Z, Denton M, Mutti N, Pappan K, Kanost MR, Reese JC, Reeck GR: **Polygalacturonase from *Sitophilus oryzae*: possible gene transfer of a pectinase gene from fungi to weevils.** *J Insect Sci* 2003, **3:**24.
