# Supplementary material for: Expression profiling and cross-species RNA interference (RNAi) of desiccation-induced transcripts in the anhydrobiotic nematode Aphelenchus avenae
Source: BMC Mol Biol. 2010 Jan 19;11:6. doi: 10.1186/1471-2199-11-6 (PMC2825203; doi:10.1186/1471-2199-11-6)
Supplement: Additional file 2 — Phenotypic analyses after RNAi treatment with ESTs listed. [file 1471-2199-11-6-S2.PDF]

CLONE	ADULTS	PRESENCE OF LARVAE	EMBRYOS	EMBRYONIC EFFECT	MORPHOLOGICAL EFFECT
EF026241	✓	✓	✓	X	X
EF026242	✓	✓	✓	X	X
GR463894	✓	✓	✓	X	X
GR463895	✓	✓	✓	X	X
GR463896	✓	✓	✓	X	X
GR463897	✓	✓	✓	X	X
GR463898	✓	✓	✓	X	X
GR463899	✓	✓	✓	X	X
GR463900	✓	✓	✓	X	X
GR463901	✓	✓	✓	X	X
GR463902	✓	✓	✓	? (late stage embryos not frequent)	X
DR121003	✓	✓	✓	X	X
GR463904	✓	✓	✓	X	X
GR463907	✓	✓	✓	X	X
GR463910	✓	✓	✓	X	X
GR463913	✓	✓	✓	X	X
GR463914	✓	✓	✓	X	X
GR463919	✓	✓	✓	X	X
GR463921	✓	✓	✓	X	X
GR463927	✓	✓	✓	X	X

Additional File 2.

Phenotypic analyses after RNAi treatment with ESTs listed.
