# Supplementary figures and images for: Expression profiling and cross-species RNA interference (RNAi) of desiccation-induced transcripts in the anhydrobiotic nematode Aphelenchus avenae
Source: BMC Mol Biol. 2010 Jan 19;11:6. doi: 10.1186/1471-2199-11-6 (PMC2825203; doi:10.1186/1471-2199-11-6)

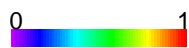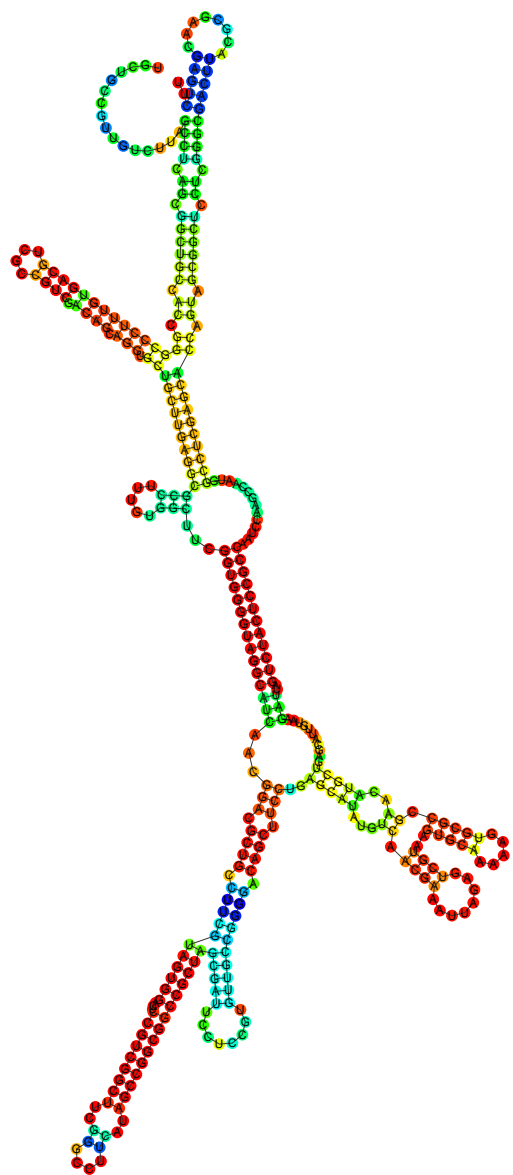

Supplement: Additional file 3 — Predicted secondary structure of RNA represented by EST GR463921 using RNAfold at http://rna.tbi.univie.ac.at with default settings. The colour scale indicates likelihood of structure formation. [file 1471-2199-11-6-S3.PDF]
