# Supplementary material for: Expression profiling and cross-species RNA interference (RNAi) of desiccation-induced transcripts in the anhydrobiotic nematode Aphelenchus avenae
Source: BMC Mol Biol. 2010 Jan 19;11:6. doi: 10.1186/1471-2199-11-6 (PMC2825203; doi:10.1186/1471-2199-11-6)
Supplement: Additional file 4 — Primer sequences used for quantitative real-time PCR of ESTs (see Tables 1 and 2). [file 1471-2199-11-6-S4.PDF]

Name	Sequence	Name	Sequence
EF026240_F	AAGGAGGAGCACAAGGTCAA	EF026240_R	CTCCTGCAGGGACTCCTTC
EF026241_F	GGTGAAGGATGCCTACGAGA	EF026241_R	CTCCTCGCGGTACTCATGTC
EF026242_F	TTCATAGCGATCAGCAGTGG	EF026242_R	AGTGCCTTTCGTTCTGGCTA
EF026246_F	AATGGGTCTCTTCGGTCACA	EF026246_R	TGTTCCTCATGGTGGTGATG
GR463894_F	TGTTGATGTCCTGCGGATAG	GR463894_R	GACCAGCAACGACTACGTCA
GR463895_F	AGCACGACGTTCGAGTAGGT	GR463895_R	ACGTTCGAACAACATCGTCA
GR463896_F	CTGTCCGACAATCACGCTAA	GR463896_R	ACAAGTGGACCTTCCCACAG
GR463897_F	CGGGAAATTCTCGCTCTACA	GR463897_R	TCGAAGCTGAAGGTGATGTG
GR463898_F	TACAACCACTTGGGCAACAA	GR463898_R	GCTCTTGCCATCCTCGTAGA
GR463899_F	TAACGTTCGCTTTTGTGTCG	GR463899_R	CCAGAGATCGGCTATGAAGG
GR463900_F	CCACAGCCATTCTTTGAGGT	GR463900_R	TATTCATCGGCTCGTCCTTC
GR463901_F	GCCAAGGACCATCTGAACAT	GR463901_R	ACGTTGCTAACAGGCTCCAT
GR463902_F	GAAACTGCACCCGTTGAGAT	GR463902_R	AACGGGTTTCAACAGTACGC
GR463903_F	TCAGCGATCATTCTTTGTGC	GR463903_R	CCTGGATCTTCGTCTTCTGC
GR463904_F	CTCTTTGCGTCAACTTCGTG	GR463904_R	TCCGAGTTCACGAGCTTCTT
GR463905_F	CTCTTCTTCCGCTGGAGCTA	GR463902_R	CTCCACCACGTCGTCAAGTA
GR463906_F	CGACAACTATGGGCGATACC	GR463906_R	CACGTATCCAACGAGCCTCT
GR463907_F	GCCAAGCCTTGAACACTCTT	GR463907_R	CGTGCAGAAGGACATAACCA
GR463908_F	CGGAAACCAGAACATCAAGG	GR463908_R	TGTCTTTGTCGGTCAGCTTG
GR463909_F	TCTCCTCGACGTCGTTAGGT	GR463909_R	AGTCGGAGCTCTCCTCCTTC
GR463910_F	TCGCAGACCTCGAAGAAGTT	GR463910_R	AACTCGTACGGGTCGAAGGT
GR463911_F	TCGGCAACAACCTTAAGTCC	GR463911_R	CCTCCTTAAGACGGGTGTCA
GR463912_F	CTCGTCAAGCTGTACGACCA	GR463912_R	TGTTCAGCTTAGGCGTGATG
GR463913_F	AGCTCGACTTGGACTTCTGC	GR463913_R	CAGCTGACGCTGGTTGATTA
GR463914_F	CCAATTTCGCAAAATGACCT	GR463914_R	GCTTGCCCTTGTACTTGTCC
GR463915_F	CCGTCGCCTCTTAAAAACAA	GR463915_R	TCAAGACGGACGAGGAAGTC
GR463917_F	AAATTGTGGAACCGAAAACG	GR463917_R	GCTCCCACCACTGATTGTCT
GR463918_F	AAGGGGTGCTTCAAATCCTT	GR463918_R	AGGCCTTTTGGTGACATACG
GR463919_F	CGCTGGAGGAACTGAAGAAC	GR463919_R	CAAGGACCTTCTCCTTCACG
GR463920_F	ACTCCTCGTGCTTCAAGGAA	GR463920_R	TTTGGGGTCAATAAGCCAAG
GR463921_F	CTGCCGTTGTCTTAGCCTCA	GR463921_R	GACGGAGTCCACTACGAAGG
GR463922_F	GTATGACAGCCGGTGAAGGT	GR463922_R	ACGCTGCTGAGTGGTTTTCT
GR463923_F	AGTCAGTCTTCGACGCACCT	GR463923_R	CGAGAGAGGAACACGTAGGC
GR463924_F	CACGCCACTGTGAACACTCT	GR463924_R	GAGCCGCTAACAATTCTCCA
GR463925_F	TGGAGGAGAAGGAGGAGGTT	GR463925_R	GTACGGTACGGGTGTGGTTT
GR463926_F	AGCACTCCACGAAGAGCAAT	GR463926_R	TGGAGACTGCATCAAAGGTG
GR463927_F	ACCTCAGTTCGTTCGGCTAA	GR463927_R	TTGAAGCTGATGGAGGCTTT
GR463928_F	TACTCGGCGTTCTTCTCCTC	GR463928_R	AGTGCCAGTGCCTTTAGTGC
GR463929_F	ACACAGCTAACCTGGCCATT	GR463929_R	AAGTGGTCCGTCTTGTCCAG
DR121003_F	TGACTTCAGATTGGGGGAAG	DR121003_R	AAGTTGCATCAGCTGCAGAG
DR121009_F	CGGAAGTTGTACTGCGTGAA	DR121009_R	GATTACGCCGCAACTAGGAA

Additional File 4.

Primer sequences used for quantitative real-time PCR of ESTs (see Tables 1 and 2).
